# Wood’s Medical and Surgical Monographs

**Published:** 1891-01

**Authors:** 


WOOD'S MEDICAL AND SURGICAL MONOGRAPHS, consisting of Original Treatises and Reproductions in English of Books and Monographs selected from the latest literature of foreign countries, with illustrations, etc. Volume VIII. Number 1, October, 1890. Number 2, November, 1890. Number 3, December, 1890. Published monthly. New York: William Wood & Company, 56 and 58 Lafayette place. 1890. Price, $10.00 a year; single copies, $1.00.

The first number in Volume VIII. of this noted series of monographs, issued in October, 1890, contains the following interesting papers: Suppuration and Septic Diseases, by W. Watson Cheyne, M. B.; Pharmacopeia for Diseases of the Skin, by James Starlin; The Nasal Neuroses, by Granville Macdonald, M. D.; Artificial Respiration, by Benj. W. Richardson, M. D.; The New-Born Infant: its Physiology, Hygiene, and Nourishment, by Dr. A. Auvard; The Urine in Neurotic Diseases, by Dr. Alexander Peyer.

The second number, November, 1890, contains: The Treatment of Uterine Affections by Massage, by Dr. Eugene Arendt; Cosmetics — A Treatise for Physicians, by Dr. Heinrich Paschkis; Affections of the Stomach in Diseases of the Male Organs of Generation, by Dr. Alexander Peyer.

The third number, December, 1890, has the following articles: Practical Guide to the Demonstration of Bacteria in Animal Tissues, by Dr. H. Kühne; On the Present Position of Antiseptic Surgery, by Sir Joseph Lister, Bart.; Cancer and its Complications, by Charles Edgerton Jennings, F. R. C. S., M. B.; The Treatment of Epilepsy, by Dr. Ch. Féré; Hand-book to Dr. Koch's Treatment in Tubercular Disease, by Drs. Grün and Severn. An index in this number completes the volume; and the second year of this series is even greater than the first.

